# The gut–liver axis in progressive steatotic liver disease: A focus on bile acid dysregulation

**DOI:** 10.1016/j.jnha.2025.100671

**Published:** 2025-09-04

**Authors:** Panayiotis Louca, Juan M. Pericàs, Yu Lin, Afroditi Kouraki, Olga Estévez-Vázquez, María Martínez-Gómez, M. Serra Cusidó, Joanna P. Simpson, Francisco Javier Cubero, Natalie Z.M. Homer, Ana M. Valdes, Cristina Menni

**Affiliations:** a Department of Twin Research & Genetic Epidemiology, King’s College London, London, United Kingdom; b Vall d'Hebron Institut de Recerca, Vall d'Hebron Barcelona Campus Hospitalari, Barcelona, Spain; c Liver Unit, Department of Internal Medicine, Vall d'Hebron University Hospital, Barcelona, Spain; d Department of Medicine, Universitat Autònoma de Barcelona, Barcelona, Spain; e Centros de Investigación Biomédica en Red en Enfermedades Hepáticas y Digestivas, Madrid, Spain; f Academic Rheumatology Clinical Sciences Building, Nottingham City Hospital, University of Nottingham, United Kingdom; g Department of Immunology, Ophthalmology and Eye Nose and Throat (ENT), Complutense University School of Medicine, Madrid, Spain; h Health Research Institute Gregorio Marañón (IiSGM), Madrid, Spain; i Centre for Biomedical Research, Network on Liver and Digestive Diseases (CIBEREHD), Madrid, Spain; j Centre for Cardiovascular Sciences, Queen's Medical Research Institute, University of Edinburgh, Edinburgh, United Kingdom; k Mass Spectrometry Core, Edinburgh Clinical Research Facility, Queen's Medical Research Institute, University of Edinburgh, Edinburgh EH16 4TJ, United Kingdom; l NIHR Nottingham Biomedical Research Centre, Nottingham University Hospitals NHS Trust and the University of Nottingham, Nottingham, United Kingdom; m Department of Pathophysiology and Transplantation, Università Degli Studi di Milano, Via Francesco Sforza, 35, 20122 Milan, Italy; n Fondazione IRCCS Cà Granda Ospedale Maggiore Policlinico, Angelo Bianchi Bonomi Hemophilia and Thrombosis Center, 20122 Milan, Italy

**Keywords:** Bile acids, Gut–liver axis, Liver disease, Gut microbiome, Metabolomics

## Abstract

**Introduction:**

The gut–liver axis regulates metabolic homeostasis, with bile acids (BAs) serving as key signalling molecules. BA dysregulation is implicated in metabolic dysfunction-associated steatotic liver disease (MASLD) and metabolic dysfunction- and alcohol-associated liver disease (MetALD), yet consistent identification of BA markers and their mechanistic roles across different stages of these diseases remain elusive.

**Methods:**

We integrated three complementary studies to examine BA dysregulation: a population-based cohort (1522 females from TwinsUK with serum BA and liver biomarker data), a clinical cohort (30 patients with steatotic liver disease, fibrosis stages F0-F4, and 4 controls), and rodent models (20 rats with MASLD/MetALD vs. 9 controls). BA profiles were quantified via LC–MS.

**Results:**

The primary bile acid taurocholate was consistently correlated with liver pathology: in TwinsUK, it associated with ALT (β [95%CI] 1.81 [1.27, 2.36], FDR < 0.05) both overall and when stratifying for age (<65 years, *n* = 923; ≥65 years, *n* = 599); in the clinical cohort, it was associated with F3 fibrosis (OR [95%CI] 8.56 × 10^−10^ [3.80 × 10^−13^, 1.93 × 10^−6^], FDR < 0.05); and in rodents, it was associated with MASLD/MetALD (OR [95%CI] 2.86 [1.17, 9.51], FDR < 0.05). The secondary bile acid taurochenodeoxycholate was associated with both early (F0, OR [95%CI] 13.63 [1.04, 179.17], *p* < 0.05) and advanced stages of disease (rodents, OR [95%CI] 15.41 [2.94, 311.82], FDR < 0.05).

**Conclusion:**

Taurocholate and taurochenodeoxycholate emerge as consistent BA markers across liver disease stages, suggesting BA metabolism as potential therapeutic targets. This multi-model study bridges knowledge gaps in BA-driven mechanisms, informing personalised strategies for SLD management.

## Highlights

• Integrates population, clinical, and rodent data for robust findings.
• Multi-model approach validates bile acid dysregulation in liver disease.
• Taurocholate links to liver pathology in all models studied.
• Taurochenodeoxycholate marks early and advanced liver disease.
• Bile acid metabolism identified as potential biomarker and therapeutic targets.

## 1 Introduction

The gut–liver axis represents a bidirectional communication system that regulates metabolic homeostasis and influences disease progression, including inflammaging and frailty [1,2]. Within this axis, bile acids (BAs) serve as essential signalling molecules, functioning both as digestive surfactants and metabolic regulators [1]. Disrupted BA homeostasis drives and results from liver disease progression. Importantly, ageing is accompanied by physiological changes in liver function, gut microbiota composition, and bile acid metabolism, which may predispose older adults to BA-related liver dysfunction [2,3].

In steatotic liver disease (SLD) and rodent models of liver injury, shifts in primary and secondary BA profiles coincide with gut microbial changes [4,5]. Three patterns emerge: (i) elevated specific BAs correlate with liver damage, (ii) primary-to-secondary BA ratios shift with disease severity, and (iii) these alterations align with microbial alterations. Fibrosis, a hallmark of chronic liver disease, progresses from stage 0 (none) to stage 4 (cirrhosis), providing a standardised measure of liver injury severity. These dynamics highlight BAs as potential biomarkers and therapeutic targets. Indeed, our prior work identified isoursodeoxycholate, a secondary BA, as a marker of cardiometabolic risk, linked to hepatic steatosis and liver enzymes [6]. Targeting BA metabolism holds therapeutic potential, exemplified by ursodeoxycholic acid, which reduces BA pool toxicity and inflammation [7].

This study builds on these findings, integrating population-based and clinical cohorts with rodent models to examine BA dysregulation across liver disease contexts. This multi-faceted approach enables cross-validation, aiming to uncover consistent BA markers and therapeutic targets. Given the interplay between aging, bile acid regulation, and liver resilience, this work provides critical insights into how age-related changes may amplify susceptibility to liver disease. By exploring BA metabolism and liver pathology interplay, we seek to identify shared mechanistic pathways and common bile acid alterations across progressive liver disease phenotypes to deepen our understanding of BA-driven mechanisms.

## 2 Methods

**Study design**: This multi-model study integrated three complementary approaches – a population-based cohort, a clinical cohort, and rodent models to examine BA dysregulation in liver disease, enabling cross-validation of consistent BA markers across paradigms. This design enabled triangulation across distinct yet complementary models, capturing subclinical variation in the general population, clinical staging in patients with SLD, and mechanistic insights in rodents, enhancing confidence in shared BA associations.

### 2.1 Human populations

**Population-based cohort:** Participants from the TwinsUK Registry [9] (1522 females, mean age 58.4 ± 14.4years, body mass index (BMI) 25.8 ± 5.1 kg/m^2^), provided informed written consent, with ethical approval from the St. Thomas’ Hospital REC (REC Ref: EC04/015).

Serum BA levels were quantified via untargeted LC–MS/MS by Metabolon Inc. (see [6]). Clinical biomarkers of liver function – alanine transaminase (ALT), a sensitive indicator of liver cell injury, aspartate transaminase (AST), a marker of hepatocellular damage, total bilirubin (elevated in liver dysfunction), and urea (reflecting liver synthetic capacity and protein metabolism) – were assessed in serum samples collected within 12 months of metabolomics profiling [10]. We also undertook a sensitivity analysis stratified by age (<65 years, *n* = 923; ≥65 years, *n* = 599) to explore whether associations between BAs and liver biomarkers were consistent across strata.

**Fig. 1:**
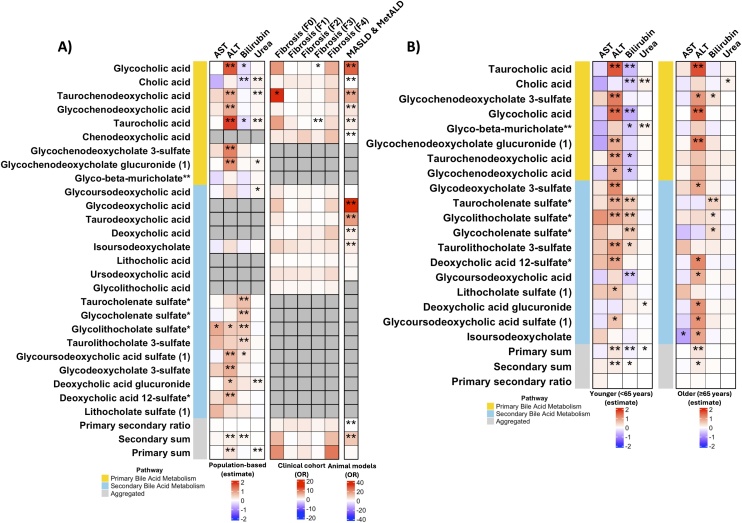
Heatmap of associations between BAs and liver pathology outcomes. **A)** Associations across population-based, and clinical human cohorts and animal models. **B)** Associations in the population-based cohort stratified by age (≥65 years; lts <65 years). The colour scale indicates the magnitude and direction of associations. ** denotes FDR < 0.05, and * *p* < 0.05. Abbreviations: AST, aspartate aminotransferase; ALT, alanine aminotransferase; MASLD, metabolic dysfunction-associated steatotic liver disease; MetALD, metabolic dysfunction- and alcohol-associated liver disease.

**Clinical cohort:** Participants with SLD were recruited from the Vall Hebron University Hospital, Barcelona, Spain – (IRB approval PR(AG)388/2021) [11], including 4 controls and 30 outpatients (56.2% female, mean age 56.3 ± 14 years, BMI 33.1 ± 8.4 kg/m²) across fibrosis stages F0–F4 (Fig. 1A). Plasma BA levels were quantified, using a targeted LC–MS/MS method that profiled 14 BAs [12].

### 2.2 Animal models

Rodent models were used to investigate BA dysregulation in metabolic liver disease. We included 10 rats with metabolic dysfunction-associated steatotic liver disease (MASLD), 10 rats with metabolic dysfunction- and alcohol-associated liver disease (MetALD), and 9 healthy controls. As there were no statistically significant differences in individual bile acid levels between MASLD and MetALD (*p* > 0.05, Wilcoxon rank-sum), these groups were combined for comparisons against controls. Furthermore, the MetALD rat model was induced with low-dose alcohol (10% ethanol), representing mild exposure that falls within the same clinical and pathophysiological spectrum as MASLD [13].

### 2.3 Statistical analysis

All statistical analysis was conducted in R (version 4.4.1) [8].

Statistical comparisons were performed using a combination of linear mixed models, multinomial regression, and Firth’s penalized logistic regression, depending on cohort structure and data type. In the population-based cohort, linear mixed models were used to evaluate associations between individual BAs and liver function biomarkers (ALT, AST, bilirubin, urea), adjusting for age and BMI as fixed effects and family relatedness as a random effect to account for twin structure. In the clinical cohort, associations between BA levels and liver fibrosis stages (F0–F4) were assessed via multinomial logistic regression, with healthy controls as the reference, adjusting for age, sex, and BMI. In rodent models, Firth’s penalized logistic regression was used to handle small sample sizes and separation bias in modelling the odds of liver disease (MASLD/MetALD) versus controls based on BA levels. False discovery rate (FDR) correction was applied across all models to account for multiple comparisons.

## 3 Results

The descriptive characteristics of the three study samples are included in Table 1.

**Table 1:** Cohort demographics and clinical characteristics.

	Population-based (TwinsUK) (*n* = 1522)		Clinical cohort (*n* = 34)		Animal models (*n* = 29)	
	Mean	SD	Mean	SD	Mean	SD
Age, years	58.4	14.4	56.3	14.0	–	–
BMI, kg/m^2^	25.8	5.1	33.1	8.4	–	–
AST, U/L	10.0	5.0	–	–	–	–
ALT, U/L	19.2	9.5	–	–	–	–
Bilirubin, μmol/L	9.1	4.6	–	–	–	–
Urea, mmol/L	5.1	1.6	–	–	–	–

	*n*	%	*n*	%	*n*	%
Sex, female	1522	100	18	52.9	–	–
Healthy controls	–	–	4	11.8	9	31.0
F0	–	–	5	14.7	–	–
F1	–	–	5	14.7	–	–
F2	–	–	5	14.7	–	–
F3	–	–	5	14.7	–	–
F4	–	–	5	14.7	–	–
F5	–	–	5	14.7	–	–
MASLD	–	–	–	–	10	34.5
MetALD	–	–	–	–	10	34.5

Abbreviations: BMI, body mass index; AST, aspartate transaminase; ALT, alanine transaminase; MASLD, metabolic dysfunction-associated steatotic liver disease; MetALD, metabolic dysfunction- and alcohol-associated liver disease; SD, standard deviation.

A heatmap summarising associations between BAs (*n* = 26) and liver disease outcomes across the population-based cohort, clinical cohort, and rodent models is presented in Fig. 1**A**.

### 3.1 Cross-cohort bile acid markers

Several BAs emerged as consistent markers across multiple cohorts demonstrating the value of a multi-model framework for exploring shared BA associations with liver pathology.

**Taurocholate**, a primary BA, showed significant associations across all models. In the population-based cohort, taurocholate positively correlated with ALT levels (β [95%CI] 1.81 [1.27, 2.36], FDR < 0.05) both overall and in individuals <65years and individuals ≥65years (Fig. 1B), weakly with urea (β [95%CI] 0.10 [0.02, 0.18], FDR < 0.05), and negatively with total bilirubin (β [95%CI] −0.29 [−0.55, −0.04], *p* < 0.05), after covariate adjustment. In the clinical cohort, taurocholate was associated with F3 fibrosis (OR [95%CI] 8.56 × 10^−10^ [3.80 × 10^−13^, 1.93 × 10^-6^], FDR < 0.05). In rodent models, it positively correlated with MASLD/MetALD (OR [95%CI] 2.86 [1.17, 9.51], FDR < 0.05).

These findings support taurocholate’s involvement in liver pathology, possibly through enhanced sodium-taurocholate cotransporting polypeptide (NTCP)-mediated uptake, consistent with studies showing increased taurocholic acid trafficking and hepatic stellate cell activation in advanced fibrosis [14].

**Glycocholate**, another primary BA, exhibited similar trends, including consistent associations in age stratified analyses, but lacked a significant association with urea levels, and its correlation with F3 fibrosis did not survive multiple testing correction. This aligns with recent reports identifying glycocholic acid as an independent risk factor for incident liver cirrhosis, though the underlying mechanisms – potentially involving metabolic acidosis – remain unclear [15].

**Taurochenodeoxycholate**, another primary BA, was positively associated with ALT (β [95%CI] 0.88 [0.31, 1.45], FDR < 0.05) overall and in individuals <65 years, but not in those ≥65 years, though the direction of effect was consistent, and urea levels (β [95%CI] 0.10 [0.02, 0.18], FDR < 0.05) in the population-based cohort and with MASLD/MetALD in rodent models (OR [95%CI] 15.41 [2.94, 311.82], FDR < 0.05). In the clinical cohort, it showed a nominally significant association with F0 fibrosis compared to healthy controls (OR [95%CI] 13.63 [1.04, 179.17], *p* < 0.05). Its consistent presence across models suggests diagnostic value for detecting early-stage dysfunction and tracking progression to fibrosis and cirrhosis, building on evidence of BA-based signatures for early NAFLD detection [4]. Notably, taurochenodeoxycholic acid, typically non-toxic, may become cytotoxic when phosphatidylinositol 3-kinase-dependent survival pathways are disrupted [16].

The overlap of key BAs across models highlights their relevance in liver disease. Taurochenodeoxycholate and glycocholate may serve as early biomarkers, while taurocholate and glycochenodeoxycholate suggest therapeutic targets for BA homeostasis.

Notably, we observed strong positive correlations between the secondary BA glycochenodeoxycholic acid, a glycine conjugate of chenodeoxycholic acid, and ALT in the population-based cohort - consistent only in younger individuals, and with MASLD/MetALD in the animal model. No significant associations appeared in the clinical cohort, likely due to small sample size and disease variability. Several secondary BAs linked to ALT and/or bilirubin in the population-based cohort were absent in the clinical cohort and animal models. This may reflect differences in BA metabolism across disease stages, cohort compositions, or methodological limitations in targeted BA detection.

## 4 Discussion

This multi-model study underscores the central role of BA metabolism in liver disease progression, identifying significant BA dysregulation across population-based, clinical, and rodent models, providing insights into the gut–liver axis. These findings extend prior work linking taurine- and glycine-conjugated BAs to fibrosis, inflammation, and metabolic dysfunction [15,16], and demonstrate their translational relevance across diverse study designs.

Taurocholate’s consistent associations with liver pathology markers, including ALT and F3 fibrosis, align with its role in NTCP-mediated uptake and hepatic stellate cell activation in advanced fibrosis [14].

Glycocholate and taurochenodeoxycholate also emerged as reliable markers, with the latter’s association with F0 fibrosis indicating a possible protective role in early disease stages, warranting further investigation. This may reflect adaptive shifts in BA composition that precede fibrogenesis and has been observed in early-stage MASLD in other cohorts [15].

In MetALD, BA dysregulation is closely tied to cholestasis-impaired bile flow and BA accumulation in the liver, which may act as both a consequence and driver of alcohol-induced injury [17]. While cholestatic features are observed in advanced SLD with overlapping metabolic and alcohol-induced liver injury [18,19], suggesting shared pathophysiological mechanisms. Additionally, ductular reaction, an adaptive response to BA-induced damage involving bile duct epithelial cell proliferation, may exacerbate fibrosis in SLD [20]. As potent signalling molecules, accumulated BAs could activate pro-inflammatory and profibrotic pathways, further promoting liver damage.

These pathophysiological links provide a rationale for targeting BA metabolism in therapeutic strategies and underscore the need to account for disease aetiology when interpreting BA profiles. The ageing process may intensify these mechanisms, as hepatic regeneration capacity, immune responses, and gut microbial diversity declines. This may partially explain increased vulnerability of older adulted to liver-related complications driven by BA imbalance [2].In age-stratied analyses, fewer BAs were linked to liver biomarkers in individuals aged ≥65, while associations were widespread but generally weaker in those <65. This may reflect subtle age-related changes in liver function or gut–liver interactions. These findings suggest that age is an important factor to consider when interpreting BA associations. Future studies should investigate the interplay of alcohol, BA metabolism, and the ductular reaction to refine disease-specific mechanisms and biomarkers. Additionally, several BAs appeared to be cohort-specific, warranting further exploration in larger, more diverse studies to clarify their role in liver disease progression.

Several limitations must be considered, including the observational nature of human data, which limits causal inference, and the TwinsUK cohort’s female-only composition, which may reduce generalisability due to sex differences in BA metabolism. This is particularly relevant given emerging evidence of sex-specific regulation of BA synthesis and transport in MASLD [21,22]. The small clinical cohort for early fibrosis stages and the translational gap between rodent models and human disease complexity necessitate larger, longitudinal studies with multi-omics integration to validate findings and explore causality.

Future research should focus on interventional studies targeting BA pathways or the gut microbiome, alongside sex-specific and age-focussed analyses in diverse cohorts, to advance precision medicine in liver disease. These findings highlight BAs as promising diagnostic and therapeutic targets, bridging gut–liver interactions and liver pathology.

## CRediT authorship contribution statement

**Conceptualisation:** CM, AMV, JMP, FJC; **Contributed reagents/materials/analysis tools:** OEV, YL, NZMH, AK; **Analysis:** PL; **Wrote the manuscript:** PL, CM, AMV, JMP, FJC; **Revised the manuscript:** All. All authors approved the final version of the manuscript.

## Declaration of Generative AI and AI-assisted technologies in the writing process

AI was not used in the preparation of this manuscript nor its figures.

## Declaration of competing interest

The authors declare the following financial interests/personal relationships which may be considered as potential competing interests:

Ana Valdes reports a relationship with ZOE global Ltd that includes: consulting or advisory. Juan M. Pericas reports a relationship with Several pharmaceutical companies that includes: consulting or advisory, speaking and lecture fees, and travel reimbursement. If there are other authors, they declare that they have no known competing financial interests or personal relationships that could have appeared to influence the work reported in this paper.

A.M.V. is a consultant for ZOE Global Ltd. J.M.P. has received consulting and speaking fees, as well as travel support, from Madrigal, NovoNordisk, Boehringer-Ingelheim, Novartis, Gilead, and MSD. All other authors declare no competing interests.
